# MAPK ERK Signaling Regulates the TGF-β1-Dependent Mosquito Response to *Plasmodium falciparum*

**DOI:** 10.1371/journal.ppat.1000366

**Published:** 2009-04-03

**Authors:** Win Surachetpong, Naresh Singh, Kong Wai Cheung, Shirley Luckhart

**Affiliations:** Department of Medical Microbiology and Immunology, University of California at Davis, Davis, California, United States of America; Stanford University, United States of America

## Abstract

Malaria is caused by infection with intraerythrocytic protozoa of the genus *Plasmodium* that are transmitted by *Anopheles* mosquitoes. Although a variety of anti-parasite effector genes have been identified in anopheline mosquitoes, little is known about the signaling pathways that regulate these responses during parasite development. Here we demonstrate that the MEK-ERK signaling pathway in *Anopheles* is controlled by ingested human TGF-β1 and finely tunes mosquito innate immunity to parasite infection. Specifically, MEK-ERK signaling was dose-dependently induced in response to TGF-β1 in immortalized cells *in vitro* and in the *A. stephensi* midgut epithelium *in vivo*. At the highest treatment dose of TGF-β1, inhibition of ERK phosphorylation increased TGF-β1-induced expression of the anti-parasite effector gene *nitric oxide synthase* (*NOS*), suggesting that increasing levels of ERK activation negatively feed back on induced *NOS* expression. At infection levels similar to those found in nature, inhibition of ERK activation reduced *P. falciparum* oocyst loads and infection prevalence in *A. stephensi* and enhanced TGF-β1-mediated control of *P. falciparum* development. Taken together, our data demonstrate that malaria parasite development in the mosquito is regulated by a conserved MAPK signaling pathway that mediates the effects of an ingested cytokine.

## Author Summary

To date, malaria remains an enormous public health burden, especially in developing countries. New strategies, including integrated vector management in combination with current conventional malaria control efforts such as drug treatment and bednet usage, could synergistically reduce malaria transmission. However, our current knowledge of vector–host–parasite interactions is limited. For example, how mosquito innate immune responses control malaria parasite development and how blood-derived factors modulate mosquito biology remain interesting topics. In this paper, we reveal the role of MEK-ERK signaling in the regulation of malaria parasite development by an ingested blood-derived, mammalian cytokine in the mosquito host. Our results provide new insights into the host–parasite–vector relationship that could be utilized as a foundation for new strategies to reduce malaria transmission.

## Introduction

Approximately 300 to 500 million malaria cases and 1 to 3 million deaths are reported annually, with the greatest numbers of deaths occurring in sub-Saharan Africa following infection with *Plasmodium falciparum* [1]. The increasing prevalence of insecticide-resistant *Anopheles* and drug-resistant malaria parasite strains has negatively impacted malaria control. As such, alternative malaria control strategies including the development of transgenic mosquito lines refractory to malaria parasite transmission have been explored [2],[3]. Studies of mosquito innate immunity have identified potential genes and molecules that are involved in parasite killing [4]–[7]. For example, inducible expression of *nitric oxide synthase* (*NOS*) and production of toxic nitrogen oxides as well as a variety of other anti-parasite effectors in the mosquito midgut [4], [7]–[9] – a critical tissue for early parasite development – suggest that the midgut response could be targeted to enhance anti-parasite resistance. In support of this concept, published data have confirmed that genetic manipulation of the NF-κB-dependent Toll and Imd signaling pathways can alter development of the murine parasite *Plasmodium berghei* in the African malaria vector *Anopheles gambiae* [10],[11]. Manipulation of cell signaling pathways in this way can facilitate the regulation of a large number of anti-parasite effectors in concert, minimizing the likelihood that the parasite could adapt to an altered host [12].

During blood digestion in the Asian malaria mosquito *Anopheles stephensi*, crosstalk between ingested human transforming growth factor (TGF)-β1 and cells of the midgut epithelium induces responses that protect the mosquito against malaria parasite infection [13],[14]. This anti-malarial response is mediated in part by TGF-β1-inducible NOS which catalyzes the production of reactive nitrogen oxides that are toxic to the parasite [4],[7]. We have identified orthologous TGF-β receptors and Smad signaling proteins in *A. stephensi* and in *A. gambiae* [15], suggesting that conserved signaling pathways could support a functional signaling framework for immunological crosstalk between human TGF-β1 and *Anopheles* cells. However, the full complement of mosquito cell signaling pathways that are activated by TGF-β1 and how this activation controls the anti-parasite response in the mosquito remain to be determined.

Transforming growth factor-β1 is a member of the TGF-β superfamily of cytokines, which are known to regulate cell differentiation, proliferation, apoptosis and pro- and anti-inflammatory immune responses [16],[17]. Given these broad biological effects, it is not surprising that TGF-β1 is a pivotal regulator of the mammalian response to malaria parasite infection, having been described as maintaining “immunological balance” during infection [18]. In mammalian cells, TGF-β1 signaling is mediated through the type I and II serine/threonine kinase receptors. After ligand binding, the TGF-β receptor complex recruits and activates SMAD signaling proteins [19]. This activation induces translocation of the activated SMAD complex into the nucleus where it regulates gene expression [20]. In addition to the SMAD signaling pathway, TGF-β1 activates other signaling proteins, particularly the mitogen-activated protein kinases (MAPKs; [21]–[23]). The MAPK signaling cascades are comprised of three protein kinases: a MAPKKK that phosphorylates and activates a MAPKK (e.g., MEK) which subsequently phosphorylates and activates a MAPK (e.g., ERK) that can regulate transcription factor activity and gene expression. The MAPKs are serine/threonine kinase signaling proteins that are responsive to stress, inflammatory mediators, and growth factors. All three of the MAPKs, including extracellular signal regulated kinase (ERK), c-Jun N-terminal kinase (JNK) and p38 MAPK have been implicated in the mammalian innate immune response to malaria infection [24]–[26] and all three are activated by TGF-β1 [27]. Cellular activation by TGF-β1 regulates both SMAD-dependent as well as SMAD-independent MAPK-regulated transcriptional responses [22],[28],[29]. Based on these observations, we hypothesized that MAPK signaling regulates the mosquito immune response to malaria parasite infection and that TGF-β1 ingested with the blood meal finely tunes this response. We have confirmed this hypothesis and suggest that our work can synergize with current efforts to target MAPKs for human genetic and infectious diseases. Small molecule MAPK agonists and antagonists can be used to unravel MAPK regulation of mosquito innate immunity and could be adapted to specifically target the mosquito host to enhance signaling through a MAPK pathway that is critical to anti-parasite defense.

## Materials and Methods

### Reagents

Human recombinant TGF-β1 was obtained from R&D Systems (Minneapolis, Minnesota). Monoclonal anti-diphosphorylated ERK1/2 was purchased from Sigma-Aldrich (St. Louis, Missouri) and polyclonal anti-ERK1/2 antibodies were purchased from Cell Signaling Technology (Charlottesville, Virginia). Anti-phospho p38 MAPK antibody was obtained from Cayman Chemical (Ann Arbor, Michigan), anti-phospho JNK1&2 antibody from Biosource (Camarillo, California), and anti-GAPDH antibody from Abcam (Cambridge, Massachusetts). Horseradish peroxidase-conjugated polyclonal rabbit anti-mouse IgG was purchased from Sigma-Aldrich and horseradish peroxidase-conjugated goat anti-rabbit F(ab')2 fragment was purchased from Biosource International (Camarillo, California). The MEK1/2 inhibitors PD98059 and U0126 were obtained from Sigma-Aldrich and from Promega (Madison, Wisconsin), respectively. The BCA assay kit and SuperSignal West Pico chemiluminescent detection kit were purchased from Pierce (Rockford, Illinois). Human serum and erythrocytes were purchased from Interstate Blood Bank (Memphis, Tennessee). RPMI 1640 with HEPES was purchased from Gibco/Invitrogen (Carlsbad, California). All other chemicals and reagents were obtained from Sigma-Aldrich or Fisher Scientific.

### Cell culture, mosquito rearing, and mosquito feeding

The immortalized, embryo-derived *A. stephensi* ASE cell line [30] was maintained in modified minimal essential medium (MEM; Gibco, Invitrogen, Carlsbad, California) with 5% heat-inactivated fetal bovine serum at 28°C under 5% CO_2_. The immortalized, minced larvae-derived *A. gambiae* 4a3B cell line was kindly provided by Hans-Michael Muller, EMBL [31]. 4a3B cells were maintained in Schneider's medium (Invitrogen) with 10% heat-inactivated fetal bovine serum at 28°C.

For *in vivo* studies, *A. stephensi* Liston (Indian wild-type strain) were reared and maintained at 27°C and 75% humidity. All mosquito rearing protocols were approved and in accord with regulatory guidelines and standards set by the Institutional Animal Care and Use Committee of the University of California, Davis. Laboratory reared 3–5 day old female mosquitoes were allowed to feed for 20 min on reconstituted human blood meals (hereafter known as “artificial blood meals”) provided through a Hemotek Insect Feeding System (Discovery Workshops, Accrington, UK). Artificial blood meals contained washed human erythrocytes and saline (10 mmol l^−1^ NaHCO_3_,15 mmol l^−1^ NaCl, 1 mmol l^−1^ ATP, pH 7.0) with or without human recombinant TGF-β1 at 2, 20, 200, 2000 pg/mL and with or without PD98059 at 4 or 40 µM. For western blot analyses, midguts were dissected from 100 mosquitoes in each treatment and control group and processed as previously described [32]. Control mosquitoes were provided artificial blood meals supplemented with an equivalent volume of diluent. Protocols involving the culture and handling of *P. falciparum* for mosquito feeding were approved and in accord with regulatory guidelines and standards set by the Biological Safety Administrative Advisory Committee of the University of California, Davis.

### Immunoblotting

Protein extracts of ASE and 4a3B cells were prepared by collecting cells in lysis buffer as previously described [33]. Briefly, 2×10^6^ cells in 2 mL medium were plated in 12 well tissue culture plates overnight for ASE cells or 4 h for 4a3B cells. After treatment, culture media were removed and cells were washed with ice cold PBS and lysed in 100 µL cell lysis buffer (10 mM Tris-HCl pH 7.4, 1 mM EDTA, 100 mM NaCl, 1 mM NaF, 1 mM EGTA, 2 mM Na_3_VO_4_, 20 mM Na_4_P_2_O_7_, 0.1% SDS, 1% Triton X-100, 0.5% sodium deoxycholate, 1 mM phenylmethylsulfonyl fluoride, 10% glycerol, 60 µg/mL aprotinin, 10 µg/ml leupeptin, 1 µg/ml pepstatin, and 1 µg/ml calyculin A). Cell lysates were cleared at 14,000 g for 10 min and the resulting supernatants were mixed with sample buffer (125 mM Tris-HCl pH 6.8, 10% glycerol, 10% SDS, 0.006% bromophenol blue, 130 mM dithiothreitol) and heated to 95°C for 4 min. Equivalent concentrations of proteins (as measured by BCA assay) were separated on 10% SDS-PAGE polyacrylamide gels and transferred to nitrocellulose membranes (BioRad). Protein loading was also visually assessed by Coomassie blue staining. Membranes were blocked in 5% nonfat dry milk (w/v) in 1× Tris-buffered saline (TBS; pH 7.0) containing 0.1% Triton-100 (TBS-T) 1 h at room temperature. For ERK detection, membranes were incubated at 4°C overnight with 1∶10,000 mouse anti-phospho-ERK monoclonal antibody or with 1∶1,000 rabbit anti-ERK polyclonal antibody in 5% nonfat dry milk 1× TBS-T. For detection of phospho-p38 MAPK, phospho-JNK, and GAPDH [34],[35], membranes were incubated at 4°C overnight with 1∶1,000 rabbit anti-phospho p38 MAPK antibody or with 1∶1,000 rabbit anti-phospho JNK1&2 antibody, or with 1∶10,000 rabbit anti-GAPDH antibody in 5% BSA 1× TBS-0.1%Tween 20. Membranes were washed 3 times, 5 min each, and incubated with a 1∶25,000 dilution of HRP-conjugated rabbit anti-mouse IgG or with a 1∶20,000 dilution of HRP-conjugated goat anti-rabbit (Fab')2 fragment at 4°C overnight. To reveal antibody-bound proteins, membranes were incubated with SuperSignal West Pico chemiluminescent reagent for 2 min. Each membrane was exposed to blue autoradiography film (ISC Bioexpress, Kaysville, Utah). Phospho-MAPK levels and total ERK levels were measured on scanned film using a GS-800 calibrated densitometer (BioRad, Hercules, California). Levels of phospho-MAPKs in each treatment were normalized to the appropriate control group.

### Real-time quantitative PCR

Total RNAs were isolated from cultured cells or from pools of 15 mosquito midguts from each treatment and control group using Trizol reagent (Invitrogen) at 6 h to 48 h after treatment. Samples were analyzed by quantitative RT-PCR using an ABI Prism 7300 Sequence Detection System (Applied Biosystems; Foster City, California). *NOS* expression levels were normalized against S7 ribosomal protein gene expression levels as previously described [36] and are represented as the fold induction over control. Primers and probes used for amplification included: *AgNOS* forward, 5′CCTGATCGGTCCCGGTACT3′; *AgNOS* reverse, 5′AATTGGCAACATTCCACATACCTT3′; *AsNOS* forward, 5′GACCAAACCGGTCATCCTGAT3′; *AsNOS* reverse, 5′GGAATCTTGCAGTCAACCATTTC3′; *S7* forward, 5′GAAGGCCTTCCAGAAGGTACAGA3′; and *S7* reverse, 5′CATCGGTTTGGGCAGAATG3′. *NOS* probe, 6-carboxyfluorescein-CACCGTTCCGTTCGTTCTGGCA-6 carboxytetramethylrhodamine; and *S7* probe, VIC-AGAAGTTCTCCGGCAAGCACGTCGT-6-carboxytetramethylrhodamine. Complementary DNAs and amplimers were generated from sample RNAs using MuLV reverse transcriptase in TaqMan® Gold RT-PCR assays (Applied Biosystems). Thermal cycling conditions were as follows: 30 min at 50°C, 10 min at 95°C, and 40 cycles of 15 s at 95°C and 1 min at 60°C. Duplicate reactions with 100 ng template RNA were analyzed simultaneously with no template controls.

### *Plasmodium falciparum* culture, mosquito feeding and analysis of infection intensity

Cultures of *P. falciparum* NF54 were initiated at 1% parasitemia in 10% heat-inactivated human serum, and 6% washed human RBCs in RPMI 1640 with HEPES (Gibco) and hypoxanthine. At day 15–17, stage V gametocytes were evident and exflagellation was evaluated on the day prior to feeding and on the day of feeding. For our assays, 5-day old *A. stephensi* were fed on mature gametocyte culture diluted with human erythrocytes and heat-inactivated serum with or without human recombinant TGF-β1 and with or without PD98059. All treatments were added to the diluted *P. falciparum* culture immediately prior to feeding. On day 8, mosquito midguts were dissected in PBS and stained with mercurochrome for direct counting of *P. falciparum* oocysts. Mean oocysts per midgut in each treatment group were calculated from all dissected mosquitoes (including zeros for mosquitoes that contained no oocysts).

### *Plasmodium falciparum* parasite growth inhibition assay

Aliquots of a single culture of *P. falciparum* NF54 were plated in 96 well flat bottom plates in complete RPMI 1640 with HEPES, hypoxanthine and 10% heat inactivated human serum. Parasites were treated with 0.004% or 0.4% DMSO (PD98059 diluent), with 0.4, 4.0 or 40 µM PD98059, with 20 or 2000 pg/ml human TGF-β1, with PBS (TGF-β1 diluent) at a volume equivalent to the highest concentration of TGF-β1 used, with 2000 pg/ml human TGF-β1 plus 40 µM PD98059, or with 10 nM or 100 nM chloroquine phosphate (Sigma-Aldrich) for 50 hr in a candle jar. The assay was terminated by adding formaldehyde to a final concentration of 1%. Erythrocytes were diluted into PBS containing 1% Triton X-100 and were stained with 1 nM YOYO-1, a fluorescent dye that intercalates into DNA and, therefore, labels parasite-infected RBCs [37]. Infected RBCs were counted with a FACS cell sorter, Becton Dickinson (Franklin Lakes, New Jersey). Growth assays were performed in triplicate within each assay and were also replicated with independent cultures of *P. falciparum*. Relative parasite growth is represented as percentages of parasite numbers in the PBS control, which was set at 100%.

### Statistical analyses

The Kolmogorov-Smirnov test was used to determine normality (Biostat 2007, AnalystSoft, Washington, DC). Data that were normally distributed were analyzed by ANOVA (α = 0.05) for overall significance and by Student-Neuman-Keuls for pairwise comparisons using Graphpad Prism version 4.0 (San Diego, California) or Student's t-test to assess differences between experimental groups at specific time intervals. Prevalences of mosquito infection were analyzed by chi-square (α = 0.05).

## Results

### Human TGF-β1 activates ERK signaling in mosquito cells

**Figure 1:**
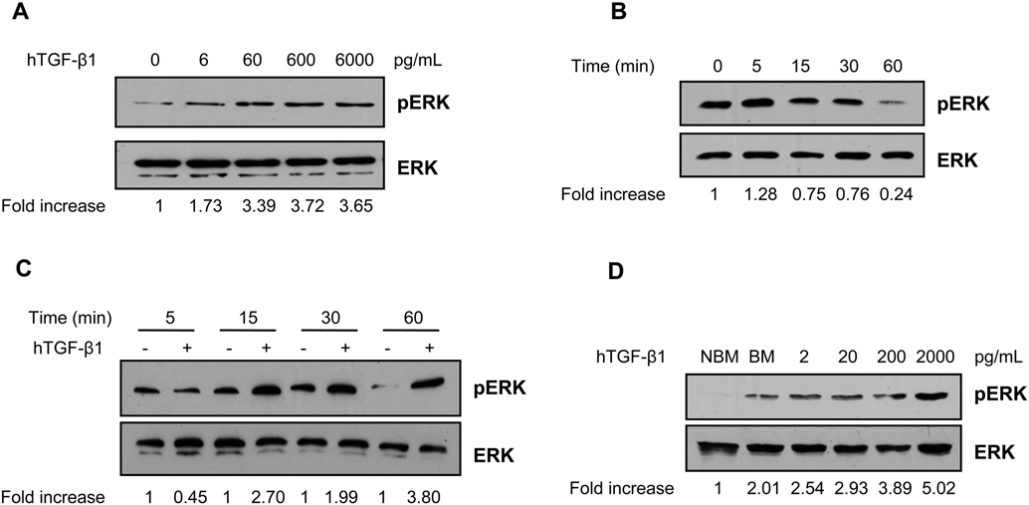
Human TGF-β1 induced ERK phosphorylation in *Anopheles* cells *in vitro* and *in vivo*. (A) *Anopheles stephensi* ASE cells were treated with PBS (0) or human TGF-β1 at concentrations from 6–6000 pg/mL. ERK phosphorylation (pERK) was examined by western blotting at 5 min after treatment. Total ERK levels provided an assessment of protein loading and were used to normalize corresponding pERK levels. The fold increases in ERK phosphorylation from densitometry analyses are indicated relative to the PBS control. (B) ASE cells were treated with 6000 pg/ml TGF-β1 for the times indicated; the 0 h timepoint indicates the pre-treatment baseline. The fold increases in ERK phosphorylation from densitometry analyses are indicated relative to 0 h baseline. (C) *Anopheles gambiae* 4a3B cells were treated with 60 pg/ml TGF-β1 or equivalent volumes of PBS for 5, 15, 30, 60 min. The fold increases in ERK phosphorylation from densitometry analyses are indicated relative to the matched PBS control within each timepoint. (D) Female *A. stephensi* were allowed to feed on an artificial blood meal supplemented with 2–2000 pg/ml TGF-β1 or with an equivalent volume of PBS as a control (BM). Midguts (n = 100) were collected and processed for protein analysis at 20 min after completion of feeding. ERK phosphorylation levels from densitometry were normalized to total ERK levels and fold inductions relative to the NBM (no blood meal) control are indicated. Figures A–D are representative of immunoblots from 2–3 independent experiments.

To determine whether ERK signaling was involved in the TGF-β1-induced anti-malarial response in mosquito cells, we assayed two *Anopheles* mosquito cell lines – ASE and 4a3B – for their response patterns. Cells were treated with doses of active human TGF-β1 that are in the range of concentrations (low pg/mL to low ng/mL) that have been detected in *P. falciparum*-infected patients [38]–[41], that bracketed blood-derived concentrations that we have detected in *A. stephensi* [13] and that we have analyzed for *NOS* induction patterns in the mosquito midgut [14]. Lysates from treated cells were analyzed by western blotting for phospho-ERK and total ERK levels. In ASE cells, human TGF-β1 dose dependently activated ERK phosphorylation (Figure 1A). Following TGF-β1 treatment, ERK phosphorylation was evident within 5 min, but was reduced at 15 min and below basal levels at 60 min after treatment (Figure 1B). In 4a3B cells, 60 pg/mL TGF-β1 maximally induced ERK phosphorylation at 15 min and activation persisted for at least 60 min after treatment (Figure 1C).

Based on our *in vitro* results, we sought to determine whether human TGF-β1 could induce ERK activation *in vivo* in the mosquito midgut. Five day old female *A. stephensi* mosquitoes were fed on artificial blood meals supplemented with doses of human TGF-β1 that spanned the range of concentrations used for cell stimulation. As shown in Figure 1D, ingested human TGF-β1 dose-dependently induced ERK phosphorylation in the *A. stephensi* midgut with the highest induction observed at 2000 pg/mL TGF-β1. Interestingly, we also observed that the artificial blood meal (BM) alone induced ERK phosphorylation up to 2-fold relative to non-bloodfed insects (no blood meal or NBM) suggesting that factors from human erythrocytes or perhaps blood ingestion and midgut expansion can induce MAPK signaling in mosquito cells.

### TGF-β1-induced ERK phosphorylation is MEK-dependent

**Figure 2:**
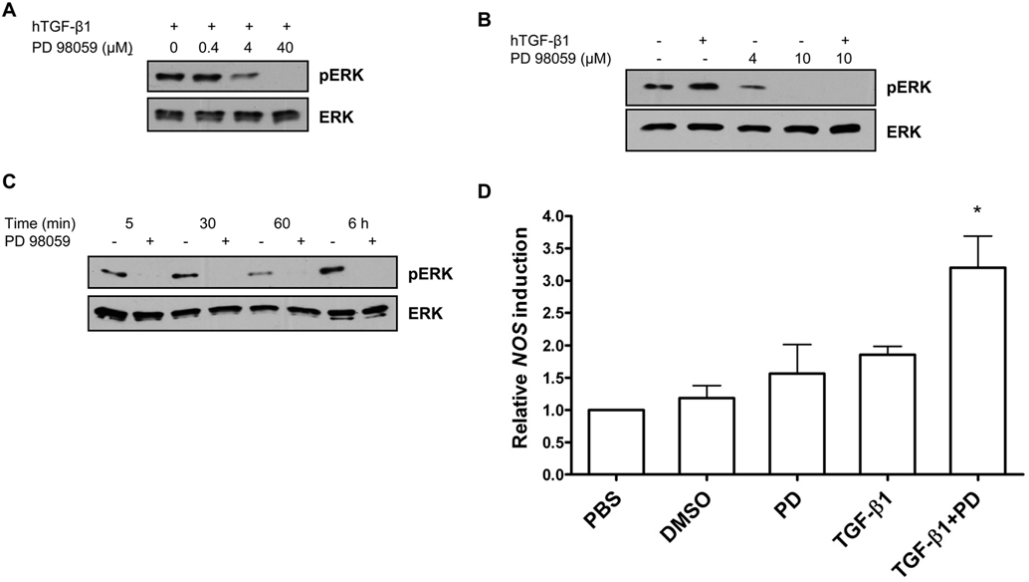
PD98059 inhibition of ERK phosphorylation increased TGF-β1-induced *NOS* expression in *Anopheles* cells. (A) The MEK1/2 inhibitor PD98059 dose-dependently inhibited TGF-β1-dependent ERK phosphorylation in 4a3B cells. Cells were pre-treated with PD98059 for 40 min then treated with 60 pg/ml human TGF-β1 for 15 min. Cell lysates were analyzed by western blotting using anti-phospho-ERK or anti-ERK antibody. (B) PD98059 inhibited TGF-β1-dependent ERK phosphorylation in ASE cells. Cells were pre-treated with PD98059 at 4 or 10 µM for 40 min then treated with 6000 pg/ml TGF-β1 for 5 min. Cell lysates were subjected to western blot analysis as in (A). (C) Inhibition of ERK phosphorylation in 4a3B cells is persistent. 4a3B cells were pre-treated with 4 µM PD98059 or with an equivalent volume of dimethyl sulfoxide (DMSO; PD98059 diluent) then treated with 60 pg/ml TGF-β1 for 5 min to 6 h. Cell lysates were subjected to western blotting analysis as in (A). Immunoblots in A–C are representative of 2–3 independent experiments. (D) PD98059 reversed the inhibitory effect of ERK activation on TGF-β1 induced *NOS* expression. 4a3B cells were pre-treated with 4 µM PD98059 or an equivalent volume of DMSO, then treated with 60 pg/ml TGF-β1. An additional control group was treated with PBS (TGF-β1 diluent) at a volume equivalent to the TGF-β1 treatment. The data are represented as means±standard errors from three independent experiments. The data were analyzed using ANOVA for overall significance and by Student-Neuman-Keuls for multiple pairwise comparisons. Legend: * = p<0.05 (TGF-β1+PD98059 versus all other groups).

To determine whether ERK activation was involved in TGF-β1 induction of *NOS* gene expression, we pre-treated cells with one of two MEK1/2 inhibitors commonly used in mammalian cell studies prior to stimulation with human TGF-β1. These inhibitors – PD98059 and U0126 – specifically inhibit MEK1/2, an upstream activator of ERK and, thus, prevent ERK phosphorylation. We first examined the effects of PD98059 on ERK activation in *A. gambiae* 4a3B cells. As shown in Figure 2A, PD98059 dose-dependently inhibited TGF-β1-induced ERK phosphorylation in 4a3B cells with complete inhibition observed at 40 µM PD98059. In ASE cells, ERK phosphorylation was notably reduced at 4 µM PD98059 and completely inhibited at 10 µM PD98059 (Figure 2B). Indeed, PD98059 potently inhibited both background ERK phosphorylation and TGF-β1-induced ERK phosphorylation in ASE cells (Figure 2B) and, in 4a3B cells, this inhibition was sustained through at least 6 h after treatment (Figure 2C).

To determine whether ERK activation by TGF-β1 regulated mosquito *NOS* gene expression, we monitored *NOS* expression in 4a3B cells that were treated with PD98059 or TGF-β1 alone at doses as in Figure 2C, pre-treated with PD98059 and stimulated with TGF-β1, or treated with PBS (TGF-β1 diluent) or DMSO (PD98059 diluent) as controls. In replicated experiments, inhibition of ERK phosphorylation increased mean TGF-β1-dependent *NOS* induction at 6 h to 3.0- to 3.2-fold relative to PBS and DMSO controls and to 1.8- to 2.1-fold relative to TGF-β1 and PD98059 alone (Figure 2D). Inhibition of ERK phosphorylation did not increase mean TGF-β1-dependent *NOS* induction at 24 h or 48 h after treatment in 4a3B cells (not shown).

To confirm that ERK activation negatively regulated TGF-β1-induced *NOS* expression, we repeated the assays with a second MEK1/2 inhibitor, U0126. Although both PD98059 and U0126 are widely used as specific MEK1/2 inhibitors, they differ in their modes of action, substrate affinity and efficacy. For example, PD98059 inhibits phosphorylation of MEK1/2 by Raf-1 [42], while U0126 inhibits MEK1/2 kinase activity directly [43]. In preliminary studies, cells were pre-treated with 2.5, 5.0 or 10 µM U0126. Reductions in ERK activation were observed with pre-treatments of 2.5 and 5.0 µM in replicated assays, but consistent inhibition was observed only at 10 µM U0126 (not shown).

**Figure 3:**
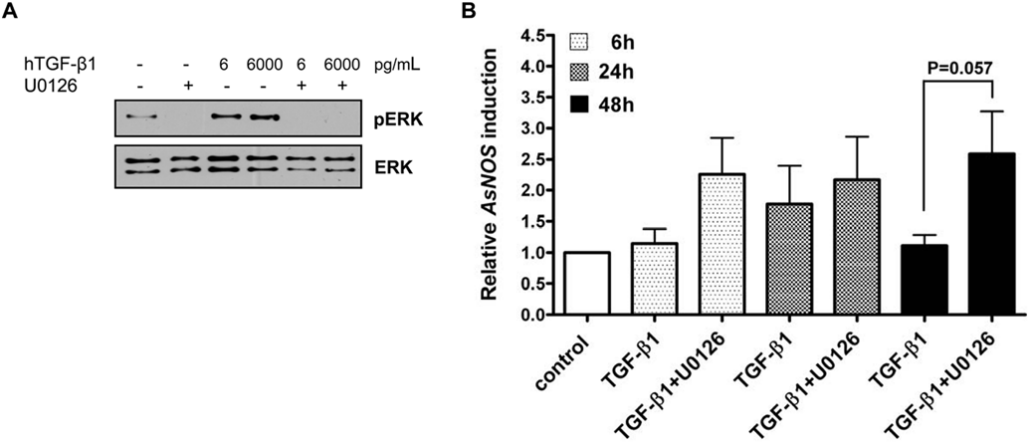
U0126 inhibition of ERK phosphorylation increased TGF-β1-induced *NOS* expression in *Anopheles* cells. (A) U0126 inhibited basal ERK phosphorylation and TGF-β1-induced ERK phosphorylation. ASE cells were pre-treated with 10 µM U0126 for 40 min then treated with 6 or 6000 pg/ml TGF-β1 or an equivalent volume of PBS. ERK phosphorylation (pERK) was examined by western blotting at 5 min after treatment; total ERK levels provided an assessment of protein loading. The immunoblot is representative of 3 independent experiments. (B) U0126 enhanced hTGF-β1-induced *AsNOS* expression. ASE cells were pre-treated with 10 µM U0126 then treated with 6 pg/ml TGF-β1 for 6, 24 and 48 h. DMSO and U0126 alone had no impact on *AsNOS* expression compared to the PBS control (not shown). *AsNOS* expression was analyzed by quantitative reverse transcriptase-polymerase chain reaction (qRT-PCR) assay. The data are represented as means±standard errors from three independent experiments for fold inductions compared with the PBS control. The data were analyzed by ANOVA for overall significance and Student's t-test was used for the pairwise comparison at 48 h.

As shown in Figure 3A, U0126 – like PD98059 – inhibited background ERK phosphorylation and TGF-β1-induced ERK phosphorylation in ASE cells. Pre-treatment with U0126 resulted in only moderate inductions at 6 h and 24 h in response to TGF-β1 relative to TGF-β1 alone (Figure 3B). By 48 h, however, when TGF-β1-induced *NOS* expression was reduced relative to the peak for TGF-β1 alone at 24 h, U0126 pre-treatment maintained TGF-β1-dependent *NOS* expression (Figure 3B).

Differences in the persistence of ERK activation (Figures 1B, 1C) and sensitivity to PD98059 (Figures 2A, 2B) noted in comparisons of *A. stephensi* and *A. gambiae* cells suggested that the differences in timing of the effects of U0126 in ASE cells (Figure 3B; 48 h) versus the effects of PD98059 in 4a3B cells (Figure 2D; 6 h) could be attributed to differences in inhibitor modes of action and efficacy as well as to species-specific differences in TGF-β1 responsiveness. Despite these differences, our results suggested that TGF-β1-dependent ERK activation was MEK-dependent and functioned to negatively regulate *NOS* gene expression in both *A. gambiae* and *A. stephensi* cells.

### ERK activation negatively regulates TGF-β1-induced *AsNOS* expression in the midgut

**Figure 4:**
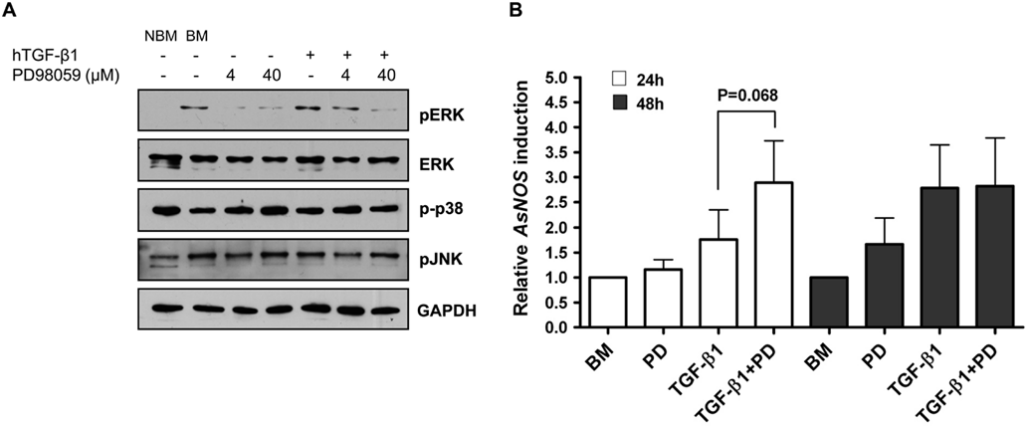
PD98059 inhibited TGF-β1-induced ERK phosphorylation and enhanced TGF-β1-induced *NOS* expression in the *A. stephensi* midgut. (A) PD98059 inhibited ERK activation in mosquito midgut. *Anopheles stephensi* mosquitoes were fed on artificial bloodmeals supplemented with 2000 pg/ml TGF-β1 with or without 4 or 40 µM PD98059. At 20 min after completion of feeding, 100 midguts from each treatment group were dissected and prepared for immunoblot analysis. Protein lysate concentrations were measured by BCA assay; equal amounts of protein were loaded in each lane. The immunoblot is representative of 3 independent experiments. (B) Inhibition of ERK phosphorylation enhanced TGF-β1-induced *NOS* expression *in vivo*. *Anopheles stephensi* were fed on artificial bloodmeals supplemented with 40 µM PD98059 (PD), 2000 pg/ml TGF-β1, or 40 µM PD98059 and 2000 pg/ml TGF-β1 (TGF-β1+PD). A control group (BM) was provided an identical unsupplemented artificial bloodmeal. Previous studies confirmed that provision of small volumes of PBS and DMSO has no effect on *AsNOS* expression *in vivo* (not shown), so these treatments were not included here. *AsNOS* expression levels were analyzed from midguts (n = 15) collected at 24 h or 48 h after blood feeding from each treatment group. The data are represented as means±standard errors from 5 independent experiments for fold inductions compared with the PBS control. The data were analyzed by ANOVA for overall significance and Student's t-test was used for the pairwise comparison at 24 h.

Based on our observations that TGF-β1 induced ERK phosphorylation in the midgut (Figure 1C) and that ERK signaling negatively regulated *NOS* gene expression *in vitro* (Figures 2D, 3B), we sought to determine whether ERK activation by TGF-β1 similarly regulated *AsNOS* gene expression in the *A. stephensi* midgut. We first confirmed that PD98059 – which significantly enhanced TGF-β1-induced *NOS* expression *in vitro* (Figure 2D) – could be used to block ERK activation *in vivo*. Five day old female *A. stephensi* were allowed to feed on artificial blood meals supplemented with PBS as a control (BM) or with 2000 pg/mL TGF-β1 with or without 4 or 40 µM PD98059. This dose of TGF-β1 was selected to assess whether a feedback inhibition of *NOS* induction *in vivo* that was attributed to NO synthesis [14] could also derive from regulatory signaling. In replicated assays, we observed that PD98059 dose-dependently inhibited ERK phosphorylation in the midgut within 20 min after blood feeding (Figure 4A). PD98059 inhibited TGF-β1-induced ERK phosphorylation with greater inhibition observed at 40 µM PD98059 (Figure 4A). Further, PD98059 action was ERK MAPK-specific – that is, phosphorylation of JNK was not affected and phosphorylation of p38 was only slightly affected by PD98059 in the *A. stephensi* midgut (Figure 4A).

To determine whether ERK activation regulated TGF-β1-induced *NOS* expression *in vivo*, we examined *NOS* gene expression in *A. stephensi* midguts at 24 and 48 h after feeding, timepoints within the range of ERK-dependent effects on *NOS* expression ASE cells *in vitro* (Figure 3B). At 24 h, ingested TGF-β1 induced *NOS* expression up to 1.7-fold compared with the PBS control group (Figure 4B). However, in the presence of 40 µM PD98059, which reduced ERK activation in the midgut (Figure 4A), TGF-β1-induced *NOS* expression was increased 1.5-fold relative to treatment with TGF-β1 alone (Figure 4B; p = 0.068). This less efficient enhancement of *NOS* induction by PD98059 *in vivo* (Figure 4B) relative to that observed *in vitro* (Figure 2D) might be due to the less efficient reduction in ERK activation by 40 µM PD98059 *in vivo* (Figure 4A) relative to that observed *in vitro* (Figure 2A). At 48 h after feeding, there was no detectable effect of PD98059 on TGF-β1-induced *NOS* expression (Figure 4B). Interestingly, we also observed a trend toward increased *NOS* expression in PD98059-treated mosquitoes relative to the PBS control group at 48 h (Figure 4B), suggesting that basal ERK activation may function to keep *NOS* expression in check *in vivo*.

### ERK activation regulates *P. falciparum* development

In a previous study, we reported that low doses (2 and 200 pg/mL) of human TGF-β1 provided by artificial blood meal reduced *P. falciparum* oocyst numbers or infection intensity in *A. stephensi*, while a high dose (2000 pg/mL) had no effect on infection intensity relative to control mosquitoes [13]. This anti-parasite effect appeared to depend in part on sustained induction of *NOS* over time at low dose compared to high dose TGF-β1 [14]. Based on our findings that inhibition of ERK phosphorylation enhanced TGF-β1-inducible *NOS* expression *in vivo* (Figure 4B), we hypothesized that ERK activation plays a pivotal role in TGF-β1-mediated control of malaria parasite development in the mosquito and that the dose-dependency of ERK activation blocks the anti-parasitic effects of TGF-β1 at the highest dose of this cytokine.

Prior to addressing these hypotheses, we sought to determine whether the effects of PD98059 were specific to the mosquito host. In our assays of PD98059 activity *in vivo*, 4 µM PD98059 reduced ERK activation, but 40 µM was required to reduce TGF-β1-induced activation of ERK to near background levels (Figure 4A). In addition, this higher dose enhanced TGF-β1-dependent *NOS* induction in the *A. stephensi* midgut at 24 h (Figure 4B). As such, we analyzed the effects of both low and high doses of PD98059 – as well as TGF-β1 and the combined treatment – using a standard *in vitro* assay of *P. falciparum* asexual stage growth.

**Figure 5:**
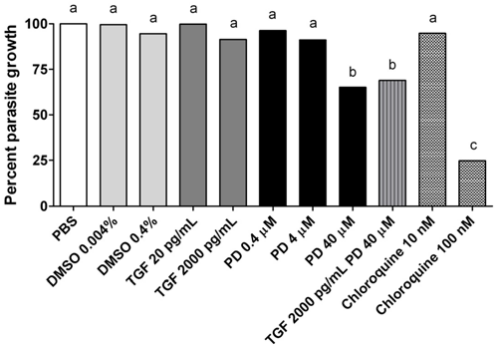
Effect of treatments on growth of asexual stage *Plasmodium falciparum*. Replicated cultures of *P. falciparum* NF54 were divided into treatment and control groups with 4 replicates per group and incubated for 50 h with the indicated treatments. The volume of PBS was equivalent to that used for TGF-β1 treatments. Parasite growth was analyzed as described in the Materials and Methods. Relative growth is compared to the PBS control which is indicated as 100%. The data are represented as means and were analyzed using ANOVA for overall significance and by Student-Neuman-Keuls for multiple pairwise comparisons. Different lower case letters indicate differences (p<0.05) among treatment groups.

In replicated assays, 10 nM chloroquine had no effect on *P. falciparum* growth relative to the PBS control, while 100 nM chloroquine significantly reduced parasite growth (Figure 5). These effects were expected and confirmed that the assay was functioning properly. Neither DMSO nor TGF-β1 at the doses tested had a significant effect on parasite growth relative to the PBS control (Figure 5). PD98059 had no effect on parasite growth at the lower doses tested (0.4 and 4 µM), but 40 µM PD98059 reduced the number of *P. falciparum*-infected RBCs relative to the PBS control. There was no difference in the effects of 40 µM PD98059 and the combined treatment (40 µM PD98059+2000 pg/ml TGF-β1) on the number of infected RBCs, indicating that the effects of the combined treatment were likely due to PD98059 (Figure 5). Because our data confirmed that inhibition of ERK activation was maximal at 40 µM PD98059 (Figure 4A) and that this dose enhanced TGF-β1-dependent *NOS* induction in the *A. stephensi* midgut (Figure 4B), we chose 40 µM PD98059 for our mosquito infection studies. Although the growth assay utilized here has not been adapted to *P. falciparum* sporogonic stages, which are metabolically distinct from asexual stages [44], we acknowledged that infection phenotypes in our studies could represent a combination of inhibitor effects on the mosquito host and on *P. falciparum*.

For our infection studies, 3–5 day old *A. stephensi* were fed on *P. falciparum*-infected erythrocytes supplemented with 2000 pg/mL TGF-β1, with 40 µM PD98059, or with both treatments. At 8 day after feeding, *P. falciparum* oocysts were counted on dissected midguts to assess infection intensity (mean oocysts per midgut) and prevalence (percentage of mosquitoes with midguts with at least one oocyst).

Infection intensity data from eight separate experiments were analyzed by two-way ANOVA for the main effects of experiment and treatment and by Student-Neuman-Keuls for means separation. Significant effects were noted for experiment (F = 68.46, df = 7, p<0.0001) and treatment (F = 11.97, df = 3, p<0.0001) as well as for the interaction of experiment and treatment (F = 6.85, df = 21, p<0.0001). These results indicated that the data could not be combined for analysis. However, means separation (α = 0.05) revealed that some data sets were not significantly different from one another and this pattern tended to reflect a separation of low and high infection intensities (Table 1). Specifically, data sets with means of fewer than 10 oocysts in the controls (PBS; Experiments 1–6) grouped together, while the data sets with infection intensity means of 17.42 and 49.50 in the controls (Experiments 7 and 8) were significantly different from the other data sets. We discuss here several trends associated with these groups.

**Table 1:** Treatment effects and trends for mean *P. falciparum* oocysts per midgut by experiment.

Mean oocysts per midgut by treatment					Trends		
Experiment	(1)PBS	(2)PD	(3)TGF	(4)TGF+PD	(1) vs (3)	(1) vs (2)	(3) vs (4)
1^a^ 1	1.70^A^ 2	1.00^A^	1.76^A^	0.24^B^	(1) = (3)	(1) = (2)	**(3)>(4)**
2^ab^	3.46^B^	6.28^A^	0.52^C^	1.88^BC^	**(1)>(3)**	(1)<(2)	(3) = (4)
3^ab^	4.75^A^	0.20^B^	2.40^A^	1.75^B^	**(1)>(3)**	**(1)>(2)**	**(3)>(4)**
4^ab^	8.14^A^	4.56^B^	7.94^A^	3.94^B^	(1) = (3)	**(1)>(2)**	**(3)>(4)**
5^ab^	9.15^A^	0.60^B^	1.25^B^	0.00^C^	**(1)>(3)**	**(1)>(2)**	**(3)>(4)**
6^b^	7.26^B^	3.36^B^	6.06^B^	12.36^A^	(1) = (3)	(1) = (2)	(3)<(4)
7^c^	17.42^A^	3.94^B^	19.22^A^	16.12^A^	(1) = (3)	**(1)>(2)**	(3) = (4)
8^d^	49.50^A^	19.28^B^	35.62^A^	15.88^B^	(1) = (3)	**(1)>(2)**	**(3)>(4)**

1 Lower case letters indicate significant differences among experiments (all data combined) by ANOVA and Student-Neuman-Keuls test (α = 0.05). For Experiments 3 and 5, a total of 20 mosquitoes were dissected per treatment group. For Experiments 1, 2, 4, 6, 7, and 8, a total of 50 mosquitoes were dissected per treatment group.

2 Upper case letters indicate significant differences among treatment groups within an experiment by ANOVA and Student-Neuman-Keuls test (α = 0.05).

At high infection intensities – which had treatment means comparable to our previous studies [13] – 2000 pg/ml TGF-β1 had no effect on infection intensity relative to the control group (Table 1; 1 vs 3 in Experiments 7 and 8) in accord with our published results. Intriguingly, however, 2000 pg/ml TGF-β1 at low infection levels significantly reduced infection intensity relative to controls in half of the data sets (Table 1; 1 vs 3 in Experiments 1–6), suggesting that control of infection by TGF-β1 may vary with *P. falciparum* oocyst load. Additional treatment differences associated with infection levels were notable. Specifically, PD98059 treatment alone decreased infection intensity at both low and high infection intensities (Table 1; 1 vs 2). And, in 4 of 6 experiments with low infection intensities and in 1 of 2 experiments with high infection intensities (Table 1; 3 vs 4), PD98059 enhanced TGF-β1-mediated control of *P. falciparum* oocyst development. Finally, at low infection intensities, the addition of PD98059 to TGF-β1 significantly increased the prevalence of uninfected mosquitoes relative to that in the TGF-β1 only treatment group in 3 of 6 experiments, with one combined treatment group completely lacking infected mosquitoes (Table 2). Despite a decrease in infection intensity in Experiment 8 (Table 1; 3 vs 4), there was no significant effect of the combination of TGF-β1 and PD98059 on prevalence of uninfected mosquitoes at this high infection intensity (not shown).

**Table 2:** Prevalences of mosquitoes without *P. falciparum* oocysts by experiment after feeding on infected blood supplemented with TGF-β1 or with TGF-β1 and PD98059.

Uninfected mosquitoes (% total dissected)				
Experiment	TGF	TGF+PD	P-value^2^	PD ↑ uninfecteds?
1^a^ 1	23 (46%)	43 (86%)	<0.0001	**YES**
2^ab^	46 (92%)	38 (76%)	0.0291	NO
3^ab^	14 (70%)	13 (65%)	NS	NO
4^ab^	19 (38%)	35 (70%)	0.0013	**YES**
5^ab^	13 (65%)	20 (100%)	0.0036	**YES**
6^b^	10 (20%)	5 (10%)	NS	NO

1 Lower case letters indicate significant differences among experiments (all data combined) by ANOVA and Student-Neuman-Keuls test (α = 0.05).

2 Two-tailed p-values calculated by chi-square test (α = 0.05).

## Discussion

In mammals, *NOS* expression is controlled by multiple signaling pathways that can activate or inhibit gene expression and enzyme activity to varying degrees (reviewed in [45]). In the context of malaria infection, TGF-β1 has been reported to regulate both early pro-inflammatory responses that function to clear parasites and later anti-inflammatory responses that function to shut down and resolve the host response to infection (reviewed in [46]). A primary target of this regulation is *NOS* [46]. Analogously, TGF-β1 also controls mosquito *NOS* expression, an important anti-parasite response in this host as well [4],[13],[14]. Specifically, low doses of TGF-β1 were observed to reduce *P. falciparum* infection in *A. stephensi*, a response that was consistent with *NOS* induction, but at high doses, there was no effect on infection [13],[14]. Feedback inhibition of *NOS* by NO at high treatment doses of TGF-β1 is partly responsible for this effect [14]. However, based on the complexity of the effects of TGF-β1 and the regulation of the multiple signaling cascades by TGF-β1, we also hypothesized that an inhibitory signaling pathway may be dose-dependently activated to reduce *NOS* expression at higher doses of TGF-β1.

Several studies prior to our work have implicated the involvement of MAPKs in mosquito innate immune responses. For example, Mizutani et al. [47] reported that a JNK-like protein was required for phagocytosis of heat-killed bacteria in *Aedes albopictus* C6/36 cells. In the same cells, Chen-Chih Wu et al. [48] reported that a p38 MAPK is required for the induction of defensin mRNA and protein after bacterial challenge. Nevertheless, no direct evidence for MAPK signaling control of malaria parasite development in *Anopheles* has been reported.

**Figure 6:**
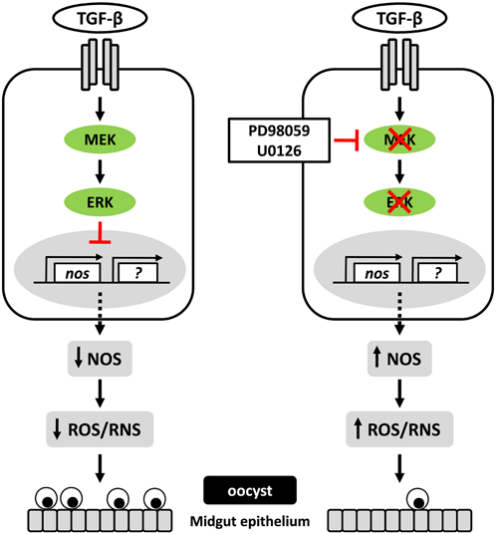
A model of MEK-ERK signaling in TGF-β1-dependent control of *P. falciparum* development. Human TGF-β1 ingested during the blood meal activates MEK-ERK signaling in mosquito cells. Activation of ERK inhibits *NOS* gene expression which reduces nitric oxide synthase levels and the synthesis of reactive oxygen and nitrogen species. MEK-ERK signaling may inhibit the expression of other anti-parasite genes as well that function together with *NOS* to limit parasite development. Inhibition of the expression of *NOS* and other anti-parasite gene products would favor *P. falciparum* development in the mosquito midgut. In contrast, inhibition of MEK-ERK signaling by the MEK inhibitors PD98059 or U0126 increases anti-parasite activity, including TGF-β1-dependent *NOS* gene expression. Increased *NOS* expression results in higher nitric oxide synthase enzyme levels and the generation of inflammatory levels of reactive oxygen and nitrogen species [7] that are toxic to the parasite.

In this study, we provide the first evidence that ingested human TGF-β1 regulates anti-parasite immunity in two different malaria vector species through a conserved MAPK module – the MEK-ERK signaling pathway. We propose in our model that MEK-ERK signaling controls TGF-β1-mediated *NOS* gene expression and *P. falciparum* development (Figure 6). Indeed, mosquitoes treated with the MEK1/2 inhibitor PD98059 and human TGF-β1 showed higher *NOS* gene expression and evidence of lower infection intensity at low infection levels or oocyst loads, suggesting that MEK-ERK signaling negatively controls mosquito innate immunity and thus favors parasite development. Inhibition of ERK activation also reduced *P. falciparum* infection prevalence in the presence of TGF-β1 to a greater degree at low infection levels, suggesting that ERK activation also tunes the mosquito host response to parasite infection. Although our present and previous works support a role for *NOS* in these events, we suggest that a suite of MAPK-regulated anti-parasite genes, some of which are TGF-β1-dependent, may collectively contribute to the control the parasite development in *A. stephensi* (Figure 6).

Under natural conditions, oocysts in field-collected *A. gambiae* rarely number more than five, indicating that the observed effects on prevalence at low infection levels would be relevant to natural conditions. Given that a single oocyst can yield an infective mosquito, only a reduction in prevalence – to near zero – can impact malaria transmission [49],[50]. Although the combined treatment at low infection levels reduced prevalence to zero in only one experiment (Table 2), we suggest based on trends here and the pace of development of MAPK inhibitors (e.g., [51]) that more specific and potent inhibition of MAPK signaling in the mosquito is possible and can yield efficient and consistent inhibition of parasite development.

Our results confirmed that P98059 and U0126 inhibit ERK activation and anti-parasite *NOS* induction in *A. stephensi* by TGF-β1 – a blood-derived cytokine that regulates *P. falciparum* infection [13] but that has no direct effects on parasite growth (Figure 5). However, the role of the parasite – and the possible effects of PD98059 on the parasite – cannot be discounted in interpreting the observed phenotypes (Tables 1 and 2). Malaria parasite infection in the mosquito is density-dependent [50] and this phenomenon likely contributes to the observed differential responses to treatment. That is, parasite factors that signal the MAPKs could be so abundant at higher infection levels as to overwhelm the effects of TGF-β1 and the combined treatment. As for the effects of PD98059 on the parasite, multiple studies have concluded that *P. falciparum* completely lacks the signaling modules for the MAPKs ERK, p38 and JNK [52],[53], but detailed studies of the effects of MAPK inhibitors on sporogonic parasites have not been completed. Despite this lack of knowledge, a reduction in parasite intensity only due to a direct effect of PD98059 on *P. falciparum* should be exhibited as proportional reductions in intensity at low and high infection levels. However, parasite intensity was reduced by 64% at high infection levels compared to 23% at low infection levels, suggesting that mosquito ERK signaling – which is likely to be proportionately greater at higher infection levels – is, in fact, contributing to the observed phenotypes.

In mammalian cells, conserved MAPK signaling pathways are critical to the regulation of anti-malarial immunity. Notably, phagocytosis of *P. falciparum* infected erythrocytes by human monocytes requires clustering of CD36 and activation of ERK and p38 phosphorylation [54]. In addition, *P. falciparum* free merozoites can inhibit human dendritic cell maturation via activation of ERK and IL-10 production [55].

The regulation of TGF-β1-induced gene transcription by MEK-ERK signaling has been reported in various mammalian cell types [22], [56]–[58]. In mammalian cells, TGF-β1 regulates gene expression through both SMAD- dependent and SMAD-independent pathways that coordinately control transcription factors and gene expression [59]. ERK activation in response to TGF-β1 can positively or negatively regulate SMAD-induced gene transcription [60]–[62]. From a mechanistic perspective, the effect of a specific blockade of the ERK pathway on mosquito *NOS* expression could be explained by the negative effect of ERK on SMAD nuclear localization and gene transcription [63],[64]. The SMAD complex is required to induce TGF-β1-mediated gene transcription and SMAD activities are regulated by several extrinsic pathways including those involving the MAPKs [22],[27]. Four MAPK phosphorylation sites have been reported in the linker region of the receptor SMADs. Mutation of these sites promotes SMAD nuclear translocation and TGF-β1-induced gene expression [61],[65]. Interestingly, a recent study revealed that a MAPK-like protein modulates bone morphogenic protein signaling and Mad-dependent gene expression in *Drosophila melanogaster* [66]. Taken together, our data suggest that MAPK regulation of TGF-β signaling is conserved in invertebrates and that the role of this regulation in innate immunity to malaria parasite infection may have deep evolutionary roots.

In mammals, TGF-β1 and the parasite factors *P. falciparum* glycosylphosphatidylinositols (GPIs) and hemozoin are key activators of the MAPKs and, hence, innate immunity during infection. In particular, parasite GPIs induce the activation of ERK, p38 and JNK and the secretion of TNF-α in murine macrophages [24]. Hemozoin enhances IFN-γ-dependent induction of *NOS* and NO synthesis through activation of ERK and NF-kB [67]. Hemozoin and *P. falciparum* GPIs also activate ERK signaling in mosquito cells [32],[33],[68]. However, ERK activation is required – and, therefore, a positive regulator – for *Anopheles NOS* induction by human insulin and by *P. falciparum* merozoites [33]. These discordant observations suggest that integration of other signaling cascades with ERK must be occurring to dictate the output of mosquito *NOS* gene expression. In mammalian cells, such pathway integration and segregation function to regulate the impact of ERK signaling [69] and are, in fact, the likely mechanisms whereby cells to respond to simultaneous, possibly divergent signals in a coordinated manner. Unraveling the likely interactions of the mosquito MAPKs with other signaling pathways – which is portended by MEK-ERK signaling complexity in mammalian cells – is a focus of our ongoing studies.

Our data have demonstrated that chemical inhibitors commonly used in mammalian cells can be applied to study signaling pathways in mosquito cells both *in vitro* and *in vivo*. The MAPKs are an intense current focus for drug development for human genetic and infectious diseases. As such, small molecule agonists and antagonists as well as immunomodulatory peptides are being developed at a pace such that we believe that the possibility exists that these molecules could be used to further unravel MAPK regulation of mosquito innate immunity. Further, peptide agonists or antagonists could be adapted in a transgenesis strategy to specifically activate or disable critical mosquito MAPK pathways to enhance anti-parasite resistance, a strategy that is of interest as a novel approach to controlling malaria parasite transmission [12].
